# Long Non-Coding RNAs in Venous Thromboembolism: Where Do We Stand?

**DOI:** 10.3390/ijms241512103

**Published:** 2023-07-28

**Authors:** Inês Soares Marques, Valéria Tavares, Beatriz Vieira Neto, Inês N. R. Mota, Deolinda Pereira, Rui Medeiros

**Affiliations:** 1 Molecular Oncology and Viral Pathology Group, Research Center of IPO Porto (CI-IPOP)/Pathology and Laboratory Medicine Dep., Clinical Pathology SV/RISE@CI-IPOP (Health Research Network), Portuguese Oncology Institute of Porto (IPO Porto)/Porto Comprehensive Cancer Centre (Porto.CCC), 4200-072 Porto, Portugal; ines.soares@ipoporto.min-saude.pt (I.S.M.); valeria.tavares@ipoporto.min-saude.pt (V.T.); i37300@ipoporto.min-saude.pt (B.V.N.); ines.mota@ipoporto.min-saude.pt (I.N.R.M.); 2 Faculty of Sciences of University of Porto (FCUP), 4169-007 Porto, Portugal; 3 Faculty of Medicine of University of Porto (FMUP), 4200-072 Porto, Portugal; 4 Abel Salazar Institute for the Biomedical Sciences (ICBAS), University of Porto, 4050-313 Porto, Portugal; 5 Research Department, Portuguese League Against Cancer (NRNorte), 4200-172 Porto, Portugal; 6 Oncology Department, Portuguese Institute of Oncology of Porto (IPOP), 4200-072 Porto, Portugal; dpereira@ipoporto.min-saude.pt; 7 Faculty of Health Sciences, Fernando Pessoa University, 4200-150 Porto, Portugal

**Keywords:** RNA, long non-coding, venous thromboembolism, deep vein thrombosis, tissue-factor-pathway inhibitor 2

## Abstract

Venous thromboembolism (VTE), a common condition in Western countries, is a cardiovascular disorder that arises due to haemostatic irregularities, which lead to thrombus generation inside veins. Even with successful treatment, the resulting disease spectrum of complications considerably affects the patient’s quality of life, potentially leading to death. Cumulative data indicate that long non-coding RNAs (lncRNAs) may have a role in VTE pathogenesis. However, the clinical usefulness of these RNAs as biomarkers and potential therapeutic targets for VTE management is yet unclear. Thus, this article reviewed the emerging evidence on lncRNAs associated with VTE and with the activity of the coagulation system, which has a central role in disease pathogenesis. Until now, ten lncRNAs have been implicated in VTE pathogenesis, among which MALAT1 is the one with more evidence. Meanwhile, five lncRNAs have been reported to affect the expression of TFPI2, an important anticoagulant protein, but none with a described role in VTE development. More investigation in this field is needed as lncRNAs may help dissect VTE pathways, aiding in disease prediction, prevention and treatment.

## 1. Introduction

The blood circulatory system (or the cardiovascular system) is responsible for transporting and delivering various compounds, including nutrients and gases, being also engaged in removing waste products [1]. As such, ensuring the integrity of the blood vessels in this complex network is vital, a function carried out by the haemostatic system, which allows continuous blood circulation [2]. 

**Figure 1:**
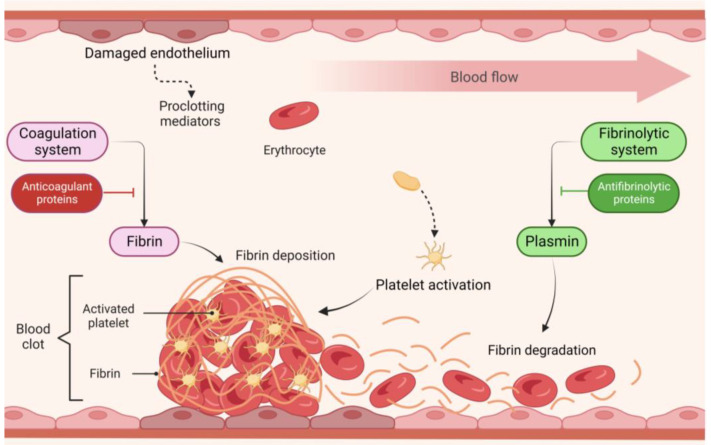
Interplay between the components of the haemostatic system. The figure was created with BioRender.com (accessed on 10 July 2023).

Haemostasis is a complex and dynamic process that rapidly responds to vascular injury. Several biochemical and cellular processes are triggered following vascular damage to form a blood clot, a process known as blood coagulation or blood clotting. This mechanism allows for the stopping of blood loss, restores haemostasis and initiates vascular repair [3]. Haemostasis is, however, under tight regulation to limit blood coagulation to sites of vascular damage, preventing haemorrhage and the formation of unnecessary blood clots known as thrombi. The generation of a thrombus (i.e., thrombosis) can be life-threatening as it might restrict or even block the blood circulation inside the vessels, preventing the transportation of compounds and exchange of gases [4,5]. Thus, proclotting and anticlotting mechanisms that regulate haemostasis must be balanced to avoid excessive bleeding and blood clotting. To maintain the haemostatic balance, four key players—vascular endothelial cells (vECs), platelets, coagulation cascade, and fibrinolytic system—interact in an intricate and tightly controlled manner (Figure 1). The damage to blood vessels activates platelets, which adhere to the exposed subendothelial matrix. Simultaneously, the coagulation cascade, a chain of enzymatic reactions that culminate in fibrin generation, is triggered to stabilize the platelet plug with fibrin and form a blood clot at the injury site to stop the bleeding [3,6]. Subsequently, the fibrinolytic system is responsible for clot lysis (i.e., fibrinolysis), which prevents thrombosis and restores blood flow. The vECs, in turn, express and/or release a set of proteins that modulate the platelet activity, the coagulation cascade and fibrinolysis towards haemostasis restoration [6]. As expected, when one of these four components is malfunctioning, the haemostatic balance can be disturbed, leading to bleeding or thrombotic disorders [5].

Deep venous thrombosis (DVT) and pulmonary embolism (PE) are two thrombotic events that collectively are known as venous thromboembolism (VTE). The former occurs when a thrombus forms within a deep vein (usually leg veins), leading to abnormal swelling and ulceration [7,8,9,10]. As for PE, this is a frequent complication of DVT that occurs when the thrombus migrates to the lungs, representing the most fatal VTE manifestation. Worldwide, every year VTE affects nearly ten million people [10]. If not treated properly, this cardiovascular disorder can be lethal, resulting in approximately 15% of mortality within the first three months following diagnosis [8,9,10]. However, even with successful treatment, the disease is also associated with a spectrum of potential complications that lead to substantial morbidity, namely VTE recurrent events, post-thrombotic syndrome, pulmonary hypertension, major bleeding under anticoagulation therapy and long-term disability [11,12]. Currently, anticoagulation therapy is the standard therapeutic approach for VTE, which, however, has proven to not always be efficient in the removal of the existing thrombus. Thus, it is crucial to dissect the molecular pathways involved in VTE pathogenesis to identify novel biomarkers and improve risk stratification, disease prevention and treatment [7,12,13].

Like other common diseases in the general population, VTE has a complex pathophysiology with both acquired and genetic risk determinants modulating the disease’s susceptibility. Advanced age, physical trauma, immobilization and active cancer constitute the most common acquired risk factors [11,14,15]. As for genetic factors, Factor V Leiden gene polymorphism (*F5* rs6025), prothrombin gene polymorphism 20210 (*F2* rs1799963), mutations causing inherited deficiencies of antithrombin, protein C and protein S, as well as the antiphospholipid antibody syndrome, account for about 7% to 22% of the elderly population attributable risk [14,15]. These factors can trigger what is known as Virchow’s triad, which encompasses three conditions that prompt VTE development, namely blood hypercoagulability, stasis of blood flow within the vessels (i.e., venous stasis) and vascular endothelial damage [16].

The most clinically used biomarker for VTE risk prediction and diagnosis is D-dimer (DD), which is a product resultant from the breakdown of cross-linked fibrin (i.e., fibrinolysis) [17]. In addition to the first VTE events, DD is also associated with the rate of recurrent events and mortality [18]. This thrombotic biomarker was first identified in the 1980s and has since been used in clinical practice for the diagnosis of VTE, particularly given its long half-life [17,19]. However, DD levels are significantly elevated in several other conditions, such as inflammation, stroke, long hospitalization periods and individuals’ advanced age, being unspecific of VTE presence [17,19,20]. To improve the specificity in detection, since DD alone is insufficient to confirm a diagnosis of VTE, elevated DD levels are followed by other diagnostic methods, including duplex ultrasonography for DVT and/or computed tomography for PE diagnosis [21,22]. Other suggested VTE biomarkers include markers for thrombophilia, for instance, soluble P-selectin and C-reactive protein (CRP). However, these biomarkers are still under evaluation and can lack specificity and/or sensitivity [23,24]. Thus, novel and trustworthy biomarkers are required to reduce the time of diagnosis and consequently improve the morbidity and mortality rates associated with VTE. Recently, emerging evidence suggested a role of long non-coding RNAs (lncRNAs) in the disease’s pathogenesis. This class of non-coding RNAs (ncRNAs) has the potential to expand our knowledge about VTE, and they represent attractive novel biomarkers of this disorder. Thus, in this narrative review, the discussion focus was the role of lncRNAs in VTE development and their implications in the haemostatic system, aiming to assess their potential clinical application as biomarkers and/or therapeutic targets in VTE management. To do so, a PubMed search without time restriction was performed using combinations of the terms “long non-coding RNA”, “lncRNA”, “venous thromboembolism”, “deep vein thrombosis”, “deep venous thrombosis”, “pulmonary embolism”, “thrombosis”, “haemostasis”, “coagulation”, “tissue factor” and “tissue factor pathway inhibitor” anywhere in the article. Relevant publications were also found by cross-referencing the bibliographic references of the retrieved papers. The identified scientific articles were published in the last six years (from 2017 to 2023). 

## 2. LncRNAs: A Novel Epigenetic Regulator in VTE

LncRNAs are ncRNAs longer than 200 nucleotides with the particularity of forming secondary structures, which enables them to bind to other biomolecules such as DNA, RNA and even proteins, thus affecting gene expression at different levels [25,26,27,28]. Another feature of these ncRNAs is the fact that they can function in the nucleus, where they are transcribed, but also at the cytoplasm, with most of them operating in both. In fact, lncRNAs can move between the two compartments in response to signals, even though the underlying mechanisms are unclear [29]. 

**Figure 2:**
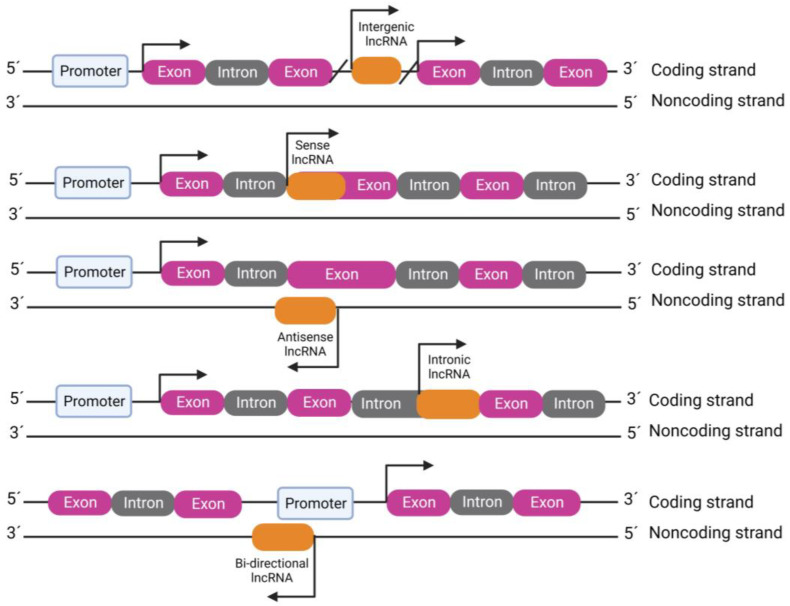
Genomic localization of lncRNAs. The figure was created with BioRender.com (accessed on 23 July 2023).

Similarly to messenger RNAs (mRNAs), lncRNAs are also transcribed by RNA Polymerase II and are often capped and polyadenylated, also presenting evolutionarily conserved patterns and a high level of tissue specificity [28]. According to the NONCODE database (version 6.0) (http://www.noncode.org, accessed on 24 May 2023), there are 96,411 human lncRNA genes [30]. Based on their genomic position, orientation and relative location to nearby protein-coding genes, these ncRNAs can be classified as intergenic, sense, antisense, intronic and bi-directional (Figure 2) [29]. Specifically, intergenic lncRNAs (lincRNAs), as the name indicates, do not overlap with any other genes [31]. As for those that do overlap, they can be sense or antisense depending on whether they possess sequence elements that will pair with other RNAs in the same or the opposite direction, respectively [32,33]. Intronic lncRNAs are located within the introns of other genes, while bi-directional lncRNAs correspond to those that are transcribed from the same region in the genome as another gene but in the opposite direction [29]. In addition to linear lncRNAs, another class is circular lncRNAs, which are formed by back-splicing of coding and non-coding transcripts [34]. 

**Figure 3:**
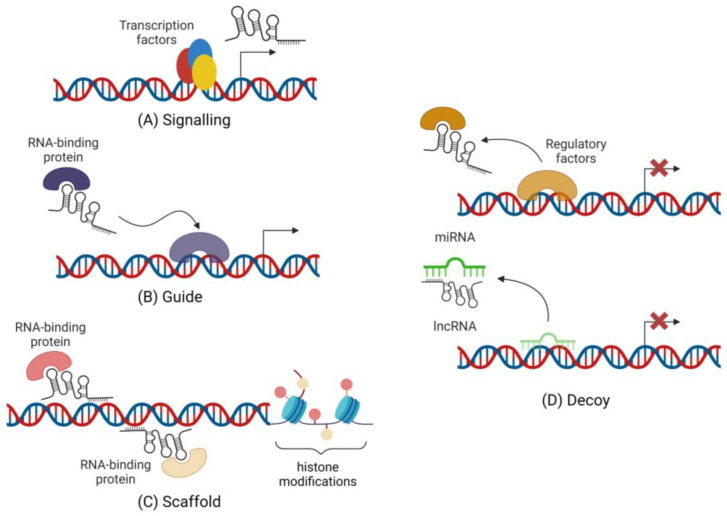
Four archetypes of lncRNAs functions. The figure was created with BioRender.com (accessed on 23 July 2023).

Despite the inconsistency in classification, the roles of lncRNAs in gene expression regulation can be divided into four main non-exclusive archetypes: signalling, decoy, guide and scaffold functions (Figure 3) [26]. As signalling molecules, lncRNAs can operate as spatiotemporal markers, reflecting the biological effects of transcription factors or signalling pathways. As decoys, lncRNAs can sequester transcription factors and other proteins into nuclear subdomains or away from chromatin. Decoy lncRNAs can also operate as competitive endogenous RNAs (ceRNAs), functioning as microRNA (miRNA or miR) sponges, consequently influencing the expression of miRNAs’ targets (mRNAs). On the other hand, guide lncRNAs can recruit RNA-binding proteins to target genes, while scaffolds may bring together several proteins to build complexes with specific biological roles, influencing transcription. Of note, lncRNAs localized in the nucleus can modify chromatin, activate or repress transcription of target genes by interacting with DNA sequences or transcription factors, while cytoplasmic lncRNAs are mostly associated with mRNA stability and translation, promoting or repressing protein synthesis [28,35,36]. Specifically, in the nucleus, lncRNAs are known to interact with DNA methyltransferases or demethylases, influencing the addition (methylation) or removal of methyl groups (demethylation) on specific genomic regions. Consequently, DNA methylation/demethylation can then lead to chromatin structure modifications [37]. Furthermore, lncRNAs can also directly mediate histone modifications of target genes. By recruiting histone modification complexes, such as histone methyltransferases or demethylases, to target gene promotor-associated binding sites, lncRNAs can induce changes in promotor histone modification patterns. These complexes then add or remove specific histone marks, such as methylation, acetylation or other modifications, on the histone tails. These modifications result in either a more open or closed chromatin conformation at the target gene promotor [38]. Both mechanisms, DNA methylation/demethylation and histone modifications, affect the accessibility of genes to the transcriptional machinery [37,38]. The versatility in these functions (chromatin regulation, transcriptional activation and repression, and RNA and protein modifications) make lncRNAs important players in normal physiology and in the development of a variety of diseases, such as cancer, cardiovascular and neurodegenerative diseases, in which their dysregulated levels have been consistently observed [8,13,28,35,39,40,41,42,43,44,45,46]. 

The first report of a putative implication of lncRNAs in cardiovascular diseases came from genome-wide association studies (GWAS). These studies independently identified single-nucleotide polymorphisms (SNPs) located in a *locus* close to the protein-coding genes CDKN2A and CDKN2B as related to susceptibility to coronary artery disease [47,48]. This *locus*, which is in human chromosome 9p21, is adjacent to the lncRNA named antisense non-coding RNA in the INK4 *locus* (ANRIL), indicating a potential role of this lncRNA in coronary artery disease, which was later corroborated by subsequent studies [49]. Recently, reports have also suggested that the expression of lncRNAs is deregulated in VTE. Interestingly, most of the reported lncRNAs were linked to the activity of vECs. As Virchow’s triad postulates, endothelial injury is a major risk factor for thrombogenesis [50]. The vECs play important roles in the maintenance of haemostasis and thrombus development [51]. In fact, several studies have confirmed the close relationship between vECs and the occurrence of DVT [52]. Under physiological conditions, these cells act as a barrier, preventing coagulation factors and platelets from activating and forming blood clots. Additionally, they secrete anticoagulant factors such as tissue factor pathway inhibitor (TFPI), nitric oxide (NO) and prostacyclin. They also exert an anticoagulant activity by expressing molecules such as heparin-like molecules, as well as thrombomodulin (TM) and endothelial protein C receptor (EPCR), both implicated in the protein C anticoagulant pathway that leads to the inactivation coagulation factors Va (FVa) and VIIIa (FVIIIa). Besides the anticoagulant activity, vECs also favour fibrinolysis via tissue-type plasminogen activator (tPA) production, which by converting plasminogen to plasmin, dissolves fibrin clots [53]. Contrarily, under pathological conditions (for instance, inflammation), vECs can express adhesion molecules such as P-selectin, which facilitates blood clot formation. Inclusively, under these conditions, these cells can become dysfunctional, which compromises their antithrombotic properties, increasing VTE risk [54,55,56,57]. 

**Figure 4:**
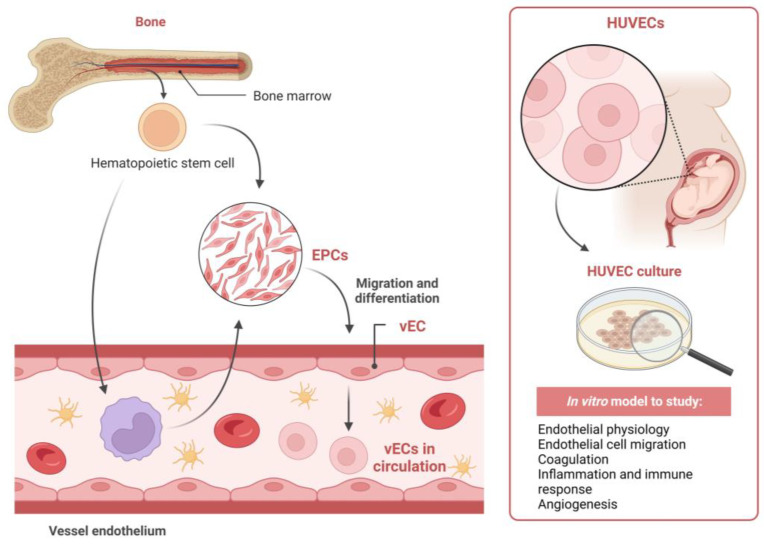
Biology of vascular endothelial cells (vECs). Abbreviations: EPC, endothelial progenitor cell; HUVEC, human umbilical vein endothelial cell. The figure was created with BioRender.com (accessed on 23 July 2023).

In addition to mature vECs, two cell populations are of relevance when it comes to dissecting the endothelium biology, namely the endothelial progenitor cells (EPCs) and human umbilical vein endothelial cells (HUVECs) (Figure 4). The former are multipotent cells derived from bone marrow that circulate in the peripheral blood and can differentiate into mature vECs when needed [13,52]. Inclusively, studies have already shown that EPCs have the potential to prevent thrombus propagation and recurrence and to promote endothelial regeneration, revascularization and vein wall remodelling [52,58]. When there is a vascular injury, a series of mechanisms are triggered to repair the damage. One of these mechanisms is angiogenesis, during which new vessels are formed from pre-existing ones, allowing the restoration of normal vascular function and thrombus resolution [53,59]. During this process, several angiogenic factors are released, such as vascular endothelial growth factors (VEGFs), fibroblast growth factors (FGFs) and platelet-derived growth factors (PDGFs), which together promote angiogenesis and stimulate the proliferation and migration of vECs to the injury site to help in the repair process [57]. Additionally, vECs can bind to platelets and inhibit their role in thrombus formation [60]. Taken together, these cells might be a promising therapeutic approach for patients for whom DVT treatments are unsuccessful [7]. As for HUVECs, they are primary cells present in the vein of the umbilical cord. These cells are commonly used as a model system for studying the biology of the endothelium and its response to environmental cues, angiogenesis and the development of cardiovascular diseases [61,62]. 

To the best of our knowledge, a total of 13 studies from 2017 to 2023 were performed, pinpointing a total of ten lncRNAs implicated in venous thrombosis (Table 1). 

### 2.1. ANRIL

ANRIL, also known as CDKN2B antisense RNA 1 (CDKN2B-AS1), is a lncRNA first identified in patients with hereditary melanoma and neural system tumour. Moreover, ANRIL is implicated in other diseases, such as atherosclerosis and cancer [63,64,65,66]. This antisense lncRNA is highly expressed in the HUVEC autophagy model. Although its roles in thrombosis are not entirely understood, ANRIL is suggested to modulate TM expression [53,67]. By cooperating with protein C and thrombin activable fibrinolysis inhibitor (TAFI), TM is important in the maintenance of the endothelial microenvironment. More specifically, TM exhibits anticoagulant and anti-inflammatory properties. Also, it enhances fibrinolysis and improves the endothelium barrier function, overall inhibiting thrombosis [53,68]. Zeng et al. [67] reported that ANRIL is highly expressed among patients with thrombosis. As having a “decoy” archetype, the authors indicated that this lncRNA might act as a ceRNA, sponging miR-99a and miR-449a in the thrombus. This mechanism consequently promotes the activation of beclin-1 while significantly increasing the levels of TM. Through these mechanisms, the authors suggested that ANRIL could promote angiogenesis and thrombosis, which seems to be conflicted. As this lncRNA was evaluated in the presence of thrombi (thrombosis patients versus healthy individuals), the data should be analysed with caution since the lncRNA might be related to thrombus resolution rather than thrombus formation. This would explain the TM upregulation. Another limitation of the study is the fact that the authors fail to differentiate the different types of thrombotic events considered in the study. To be noted, ANRIL has also been associated with different manifestations of arterial thrombosis [69,70]. Overall, more studies are needed to clarify the role of ANRIL in venous thrombosis. 

### 2.2. GUSBP5-AS

The recently identified lncRNA GUSB pseudogene 5 antisense (GUSBP5-AS) was shown to be upregulated in EPCs among DVT patients [7,13]. Acting as a decoy, this ncRNA is reported to sponge miR-223-3p, which targets forkhead box protein O1 (FOXO1). This leads to the activation of the AKT pathway, which enhances fibroblast growth factor 2 (FGF2), matrix metalloproteinase-2/9 (MMP2/9) and F-actin expression, promoting angiogenesis, EPC migration and invasion while reducing the apoptosis of these cells. As previously mentioned, these mechanisms are important for thrombus resolution and recanalization, as well as to improve the home ability of EPCs to thrombosis sites. Taken together, GUSBP5-AS seems to promote thrombus resolution and, thus, might help pinpoint a new potential therapeutic approach for DVT [7]. 

### 2.3. MEG9

The lncRNA maternally expressed gene 9 (MEG9) is suggested to protect the vasculature from DNA damage, according to an abstract. While DNA-damaging agents are known to induce the expression of this lncRNA, vascular growth factors have the opposite effect. The knockdown of MEG9 inhibits proapoptotic proteins, increases vEC death and decreases sprouting angiogenesis, implying that MEG9 might have a regulatory role in thrombosis since its inhibition in vECs accelerated fibrin formation [71]. Even though the available data about this lncRNA remains scarce, this abstract, together with other findings, indicates that MEG9 in vECs might impact pathways associated with inflammation and thrombosis [71,72,73,74]. More studies are required to explore the role of this lncRNA in VTE pathogenesis.

### 2.4. MALAT1

The lncRNA metastasis-associated lung adenocarcinoma transcript 1 (MALAT1), as the name indicates, was first identified in non-small cell lung cancer (NSCLC) and in association with lung cancer metastasis [75]. Since then, this lincRNA has been intensively studied, with reports of its deregulation in several conditions, such as cancer and Parkinson’s disease [9,76,77,78,79,80,81]. Concerning vascular disease pathogenesis, MALAT1 has been pointed out as a major player due to its downregulation in atherosclerotic plaques and its association with vEC function [8,82]. There has been a growing interest in understanding the mechanisms through which MALAT1 manages to impact DVT. The performed studies had, however, inconsistent results, which led to the hypothesis that MALAT1 may have a dual role in thrombosis. On the one hand, this lncRNA was found to be upregulated in DVT through regulation of the activity and behaviour of EPCs [8,9]. Concordantly, in the same study, silenced MALAT1 enhanced the growth, migration and survival of EPCs. Those changes were reversed when the Wnt/β-catenin signalling pathway was inhibited, suggesting that this pathway is a potential downstream target of MALAT1 in DVT. Thus, the MALAT1/Wnt/β-catenin axis might be a promising novel therapeutic target for DVT treatment [8]. In another study, MALAT1 modulated HUVEC apoptosis by regulating the miR-383-5p/BCL2-like 11 (BCL2L11) axis in DVT. When overexpressed, this lncRNA repressed miR-383-5p, which otherwise would target BCL2L11, suggesting this ncRNA as a decoy. In turn, higher levels of BCL2L11 reduced cell viability and increased vEC apoptosis. Therefore, the MALAT1/miR-383-5p/BCL2L11 axis might also be effective for DVT management [9]. A more recent study investigated the mechanisms by which MALAT1 affects platelet activity and thrombus formation, as this is one of the most abundant lncRNAs in platelets. According to the results, platelet deficiency of MALAT1 was reported to enhance platelet adhesion and aggravate thrombus generation through the phosphoinositide 3-kinase (PI3K)/AKT/Glycogen synthase kinase-3 beta (GSK-3β) signalling pathway, which contradicts the previous findings [83]. Taken together, the data indicate that MALAT1 might have a context-dependent role in DVT pathways. Additional studies are needed to better comprehend its implications in thrombogenesis, as this lncRNA seems to be a promising biomarker and therapeutic target for DVT.

### 2.5. SIRT1-AS

Sirt1 antisense lncRNA (Sirt1-AS) was first identified in 2014 and is implicated in myogenesis via the regulation of silent information regulator 1 (Sirt1) expression [84]. Sirt1 is a NAD+-dependent lysine deacetylase (member of the sirtuins family) activated in response to cellular stress. This enzyme is known to inhibit the development of several diseases, such as pulmonary fibrosis and cerebral ischemia, including also thrombotic events, by regulating the activity of platelets and vECs [85,86,87,88]. Specifically, sirt1-AS can bind and overlap with the 3′ untranslated region (3′-UTR) of Sirt1 mRNA, forming a lncRNA-mRNA duplex, acting as a guide able to increase Sirt1 stability and expression. By promoting Sirt1 expression, Sirt1-AS can suppress the development of endothelial ageing and alleviate the formation of thrombus by improving the viability and proliferation of HUVECs [37]. Concordantly, in patients with DVT, Sirt1-AS is reported to be downregulated, which may affect Sirt1 abundance leading to an increased risk of thrombotic events [37,89,90]. Overall, the current evidence suggests that Sirt1-AS could be a potential DVT biomarker [37].

### 2.6. LINC01123

Long intergenic non-protein coding RNA 1123 (LINC01123) is known to act as an oncogene [91,92,93]. This lincRNA is also reported to play a role in carotid atherosclerosis by promoting cell proliferation and migration [94]. In a recent study, LINC01123 was shown to have a relevant role in lower extremity DVT (LEDVT) in rats via the miR-125a-3p/interleukin-1 receptor type 1 (IL1R1) axis. By sponging miR-125a-3p, which targets IL1R1, LINC01123 is associated with a rise in the length and weight of the thrombus, as well as higher levels of pro-inflammatory cytokines, namely interleukin-6 (IL-6) and interleukin-8 (IL-8). This, in turn, favours inflammatory cell infiltration, thrombosis in the lumen, and collagen fibre hyperplasia, which collectively leads to thrombo-inflammation in the setting of LEDVT. The findings might give some insights into novel treatment options for patients with this condition [95].

### 2.7. TUG1

LncRNA taurine upregulated gene 1 (TUG1) was first identified as upregulated in developing retinal cells and in response to taurine (2-aminoethanesulfonie acid) [96]. This lncRNA is also implicated in other conditions, namely colorectal cancer and obesity-related diseases [97,98]. More recently, TUG1 was shown to have a poor expression in DVT mice. However, by downregulating miR-92a-3p and subsequently upregulating the 3-hydroxy-3-methylglutaryl-coenzyme A reductase (HMGCR) pathway (known to regulate angiogenesis), TUG1 overexpression was able to accelerate the proliferation, migration and tube-forming abilities of EPCs, while decreasing their apoptosis and the thrombus size. Collectively, this lncRNA, by serving as a decoy, seems to exert a protective effect against DVT and provide femoral vein pathological damage. Thus, the TUG1/miR-92a-3p/HMGCR cascade might open new perspectives to treat DVT [58]. 

### 2.8. XIST

The lncRNA X inactive-specific transcript (XIST) is one of the most studied lncRNAs, known to be required for X-chromosomal inactivation during early development for dosage compensation [99]. In addition, XIST is also associated with preeclampsia and cancer [100,101]. This lncRNA was found to be highly expressed in the plasma of DVT patients, possibly acting as a decoy by sponging miR-103a-3p, which in turn, targets high-mobility group box 1 (HMGB1) [102]. Concordantly, miR-103a-3p expression was found to be reduced in patients with unprovoked VTE [103]. In DVT, lower levels of this microRNA inhibited normal functions of EPCs, such as migration and angiogenesis [104]. On the other hand, higher miR-103a-3p expression levels, together with lower levels of chemokine 12 (CXCL12), inhibited the development of LEDVT, which is in line with previous data [105]. As for HMGB1, its levels are highly expressed in DVT patients. This nuclear protein is one of the main regulators of the coagulation cascade, also known to induce inflammatory reactions and play a part in apoptosis. In thrombosis, HMGB1 has a pro-thrombotic effect as it promotes both the activation and aggregation of platelets [102,106]. Also, via the XIST/miR-103a-3p/HMGB1 axis, XIST may regulate the expression of tissue factor (TF, also known as thromboplastin or coagulation factor III (FIII)) in HUVECs induced by IL-1β, as well as increase cell viability and hinder apoptosis by inhibiting the reactive oxygen species (ROS)/nuclear factor κB (NF-κB) signalling pathway [102,107,108,109]. 

### 2.9. LINC00659 and UXT-AS1

LINC00659, a novel lncRNA, was found highly expressed for the first time in colorectal cancer with implications in cancer cell growth and apoptosis, possibly through the PI3K-AKT pathway [110]. More recently, this lncRNA was implicated in gastric cancer [111,112]. High altitude is known to activate the coagulation system, increasing the risk of developing VTE, although there is still a lack of evidence regarding this matter. However, a pro-thrombotic phenotype often occurs, mainly in travellers and mountaineers, possibly due to the hypoxia state present at high altitudes, which increases platelet aggregation and triggers blood coagulation [113,114,115,116,117]. In this context, Jha et al. [114] aimed to compare the lncRNA expression profiles in the peripheral blood of human patients with DVT at high altitudes with control subjects without DVT. According to the results, LINC00659 and UXT antisense RNA 1 (UXT-AS1) were upregulated in DVT patients, possibly because both of these lncRNAs sponge miR-15 and miR-143, which otherwise would inhibit the expression of the pro-thrombotic genes *serpin family E member 1* (*SERPINE1*) and *hypoxia-inducible factor 1 subunit alpha* (*HIF1A*). The former encodes for plasminogen activator inhibitor 1 (PAI-1), a key inhibitor of fibrinolysis, by blocking the activation of plasminogen to plasmin [118]. On the other hand, HIF1 (encoded by *HIF1A*) regulates the activity of the NLR family pyrin domain containing 3 (NLRP3) inflammasome, a protein complex involved in the formation of blood clots under hypoxia [119]. To be noted, as UXT-AS1 has no described role in VTE other than DVT associated with high altitude, this lncRNA does not fit the scope of this review. In another study, aiming to dissect the role of LINC00659 in vECs, the lncRNA was also reported to target and downregulate miR-525-5p, which, in turn, is known to downregulate BCL2 associated X, apoptosis regulator (Bax), a member of the Bcl-2 family of proteins linked to apoptosis, suggesting its role as a decoy. Via this mechanism, LINC00659 could inhibit the proliferation and cell viability of HUVECs and promote their apoptosis. Thus, the authors revealed a potential mechanism through which LINC00659 might impact DVT and indicate the LINC00659/miR-525-5p/Bax axis as a potential new direction for the diagnosis and treatment of DVT [120]. In a more recent study aimed to unravel the role of LINC00659 in LEDVT, the lncRNA, in addition to being elevated both in inferior vena cava tissues and EPCs of patients with LEDVT, also inhibited proliferation and migration of EPCs, as well as angiogenesis [121]. As an explanation, the authors hypothesized that LINC00659 might bind to the promoter of eukaryotic translation initiation factor 4A3 (EIF4A3) to upregulate its expression, suggesting that this ncRNA acts as a guide to promote EIF4A3 expression. In turn, EIF4A3 represses fibroblast growth factor 1 (FGF1) expression and facilitates its methylation through the recruitment of DNA methyltransferases 3A (DNAMT3A) to its promoter region. As FGF1 promotes EPCs viability and neovascularization in addition to its role in cell proliferation, migration, invasion and angiogenesis, by repressing this protein, LINC00659 might increase the risk for LEDVT development [122,123]. 

### 2.10. CRNDE

Long non-coding RNA Colorectal neoplasia differentially expressed (CRNDE) was initially found to be upregulated in colorectal cancer tissues [124]. More recent studies have indicated that this lncRNA is also implicated in other malignancies, in addition to regulating vascular smooth muscle cell behaviour and inflammation and apoptosis in alcoholic liver disease [125,126,127,128]. A recent study found that CRNDE and the prenylcysteine oxidase 1 (PCYOX1) were upregulated in the blood of DVT mice [129]. The protein PCYOX1 is an enzyme involved in the breakdown of prenylated proteins found in vascular and blood cells. Deficiency of PCYOX1 is suggested to cause platelet hypo-reactivity [130,131]. Thus, acting as a decoy and through the sequestering of miR-181a-5p (targets PCYOX1), CRNDE might lead to PCYOX1 upregulation, exacerbating thrombus formation and vascular inflammatory injury in DVT [129].

**Table 1:** LncRNAs with reported roles in VTE pathogenesis.

LncRNA	Study	LncRNA Location ^1^	LncRNA Expression in VTE	Sample/Compartment or Study Model	LncRNA Target	Mechanism of Action	LncRNA Function in VTE
ANRIL	Zeng et al. (China, 2019) [67]	9p21.3	↑	HUVECs and rats	↓miR-99a ↓miR-449a	Increases beclin-1 expression via miR-99a and miR-449a sponging and upregulates thrombomodulin	Promotes thrombosis
GUSBP5-AS	Sun et al. (China, 2020) [7]	4q31.21	↑	Human EPCs and mice	↓miR-223-3p	Sponges miR-223-3p, which targets FOXO1 and activates the Akt pathway and enhances FGF2, MMP2/9 and F-actin expression	Promotes DVT resolution
MEG9	Espinosa-Diez et al. (EUA, 2020) [73]	14q32.31	n.a	ECs	n.a	Inhibits fibrin formation	Possibly has a protective effect against thrombosis
MALAT1	Du et al. (China, 2020) [8]	11q13.1	↑	Human EPCs	Wnt/β-catenin	Reduces endothelial function	Promotes DVT
	Wang et al. (China, 2022) [9]			HUVECs	↓miR-383-5p/BCL2L11		
	Sun et al. (China, 2022) [83]			CD34+ megakaryocytes and mice	PI3K/AKT/GSK-3β	Inhibits platelet activity and thrombus formation	Inhibits DVT
Sirt1-AS	Lou et al. (China, 2021) [39]	10q21.3	↓	Patients’ blood, mice and HUVECs	↑Sirt1	Suppresses the endothelial ageing and alleviates the thrombus formation through Sirt1/FOXO3a axis	Attenuates ageing-related DVT
LincRNA 1123	Yang et al. (2022, China) [95]	2q13	↑	Rats	↓miR-125a-3p	Sponges miR-125a-3p, which targets IL1R1, facilitating thrombus formation and inducing higher levels of IL-6 and IL-8	Promotes LEDVT
TUG1	Feng et al. (China, 2022) [58]	22q12.2	↓	Mice EPCs and mice	↓miR-92a-3p	Accelerates proliferation, migration and tube-forming abilities and decreases apoptosis of EPCs and thrombus size through downregulation of miR-91a-3p and upregulation of HMGCR	Protects against DVT
XIST	Cao et al. (China, 2022) [102]	Xq13.2	↑	Human plasma and HUVECs	↓miR-103a-3p	Reduces ECs normal functions through miR-103a-3p sponging	Promotes DVT
LINC00659	Zhang et al. (China, 2023) [121]	20q13.33	↑	Human IVC tissue samples, human EPCs and mice	↑EIF4A3	Upregulates EIF4A3 expression and exacerbates endothelial progenitor cell dysfunction	Promotes LEDVT
	Zhu et al. (China, 2023) [120]		n.a	HUVECs	↓miR-525-5p	Inhibits proliferation and cell viability in HUVECs through downregulation of miR-525-5p, which targets Bax	Possibly promotes DVT, given its role in HUVECs
CRNDE	He et al. (China, 2023) [129]	16q12.2	↑	Mice IVC	↓miR-181a-5p	Competitively bound to and inhibits miR-181a-5p, promoting Pcyox1l expression and aggravating thrombus formation in DVT	Promotes DVT

Abbreviations: DVT, deep vein thrombosis; ECs, endothelial cells; EPCs, endothelial progenitor cells, HUVECs, human umbilical vein endothelial cells; IVC: inferior vena cava; LEDVT; lower extremity deep vein thrombosis; lncRNA; long non-coding ribonucleic acid; n.a, not available; VTE, venous thromboembolism. ^1^ According to HGNC: HUGO Gene Nomenclature Committee (https://www.genenames.org/, accessed on 20 May 2023) and Ensembl database (https://www.ensembl.org/index.html, accessed on 20 May 2023). LncRNA or target expression: ↓ downregulated and ↑ upregulated.

## 3. LncRNAs Targeting the Coagulation System

The coagulation system plays a leading role in VTE pathogenesis. Inclusively, as previously mentioned, treatment with anticoagulants constitutes the standard therapeutic approach to treat VTE events [132]. The most used anticoagulants are low molecular weight heparin (LMWH), unfractionated heparin (UFH) and direct oral anticoagulants (DOACs). LMWH and UFH bind to antithrombin III to inhibit the activity of several clotting factors, namely thrombin (or activated coagulation factor II (FIIa)) and activated coagulation factor X (FXa). On the other hand, DOACs act by inhibiting either thrombin or FXa, depending on the drug used [133].

**Figure 5:**
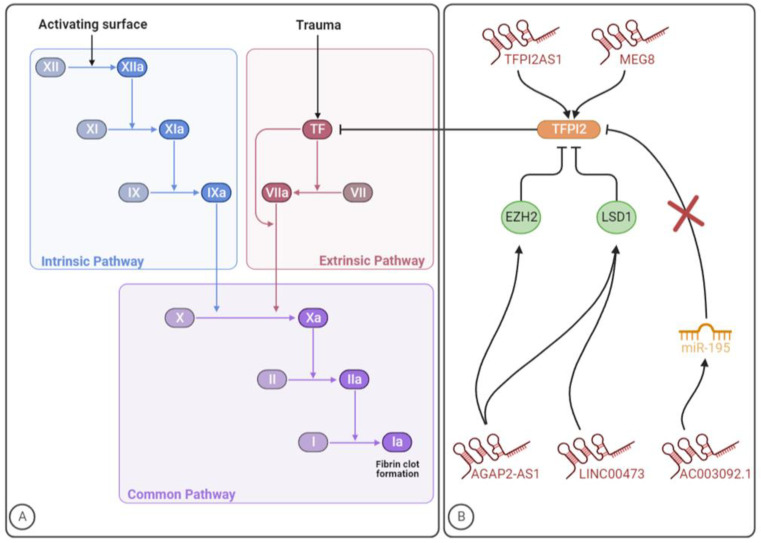
The involvement of lncRNAs in the clotting cascade. (**A**) Coagulation cascade. (**B**) LncRNAs that regulate TFPI2 expression/activity. The figure was created with BioRender.com (accessed on 10 July 2023).

The coagulation cascade involves a series of sequential proteolytic activation reactions, with a complex interplay between several proteins named coagulation factors that culminate in fibrin formation. There are three major coagulation pathways: extrinsic (also called the TF coagulation pathway) and intrinsic (contact coagulation pathway), which converge on a common coagulation pathway [134]. Thromboplastin, or TF, is a transmembrane protein located constitutively on the surface of all cells except for endothelial and blood cells. When damage to blood vessels occurs, TF is released into the bloodstream, initiating the extrinsic coagulation pathway. By combining coagulation factor VII (FVII), the complex TF-FVII (extrinsic tenase complex) activates coagulation factor X (FX) [135]. FXa then participates in the common coagulation pathway. On a phospholipid surface, FXa forms a complex with activated coagulation factor V (FVa), known as prothrombinase complex, that converts prothrombin (coagulation factor II (FII)) into thrombin. Next, thrombin transforms fibrinogen (coagulation factor I (FI)) into fibrin (activated FI (FIa)) that deposits at the vascular injury site, forming a network together with platelets to create a blood clot [136]. Parallelly, the intrinsic coagulation pathway is initiated by factors released into the surrounding damaged tissue and circulating blood after injury, such as collagen and other negatively charged surfaces. Once activated, coagulation factor XII (FXII) activates coagulation factor XI (FXI), which then activates coagulation factor IX (FIX). In the presence of calcium ions (coagulation factor IV (FIV)), activated FIX (FIXa) forms a complex with coagulation factor VIII (FVIII) that generate FXa [137]. Both extrinsic and intrinsic pathways thus produce FXa, which then participates in the common coagulation pathway leading to thrombin generation and, ultimately, fibrin deposition (Figure 5A) [136]. To be noted, thrombin also activates TAFI, FV, FVIII, FXI and coagulation factor XII (FXIII), creating a positive feedback loop that enhances clot formation. In addition, thrombin also promotes platelet adhesion by inactivating a disintegrin and metalloprotease with thrombospondin type 1 motif (ADAMTS13). On the other hand, this multifunction protein has anticoagulant activity through the activation of protein C, a natural anticoagulant that regulates the blood coagulation system through the inactivation of FVa and FVIIIa [138].

Until now, few studies have been conducted to identify lncRNAs targeting the coagulation system, which were focused on the tissue factor pathway inhibitor 2 (TFPI2). As the name already indicates, TFPI is a serine protease inhibitor that blocks the activity of the extrinsic or TF coagulation pathway [139]. Specifically, under physiological conditions, this natural anticoagulant protein is known to damp coagulation by effectively inhibiting the extrinsic tenase complex and the prothrombinase complex [140,141]. Beyond coagulation disorders, deregulated levels of TFPI were shown in several diseases, such as cancer, diabetes mellitus and renal diseases [142,143,144,145,146,147,148,149,150]. Tissue factor pathway inhibitor 1 (TFPI1) and TFPI2 are two distinct coagulation inhibitors (encoded by different genes) with the role of maintaining the haemostatic balance [151]. Alternative splicing of TFPI1 leads to the synthesis of two primary isoforms, namely TFPIα and TFPIβ. The former is a soluble protein released into the bloodstream by vECs and activated platelets, while the latter is a glycosylphosphatidyl inositol-anchored protein presented at the endothelium surface. Moreover, TFPIα inhibits both extrinsic tenase and prothrombinase, whereas TFPIβ blocks the extrinsic tenase complex more effectively, not being able to inhibit the prothrombinase complex [139]. As for TFPI2, it has inhibitory activity towards tenase complex, FXIa, plasmin and some matrix metalloproteinases [142,149]. Reduced synthesis of TFPI2 has been associated with angiogenesis, inflammation, atherosclerosis and tumour growth and metastasis [152].

To the best of our knowledge, a total of five studies from 2017 to 2022 were performed identifying five lncRNAs with implications in TFPI2 expression and/or activity (Table 2). The mechanisms through which these lncRNAs might impact TFPI2 and, consequently, the coagulation pathway, are summarized in Figure 5B. Given the role of the TF coagulation pathway in VTE development, further investigation should be carried out to explore whether lncRNAs regulating TFPI2 expression might impact the disease pathogenesis and dissect the underlying mechanisms of action.

### 3.1. TFPI2AS1

TFPI2 antisense RNA 1 (TFPI2AS1) is an antisense lncRNA that is reported to positively regulate TFPI2 expression in NSCLC tissues, acting as a guide. Specifically, TFPI2AS1 is upregulated and inhibits tumour cell proliferation and metastasis by upregulating TFPI2 expression, although the underlying mechanisms are unclear [153]. Beyond cancer-induced hypercoagulability, dysregulation of TF-associated signalling pathways and their constituents play an important role in tumour progression, particularly by impacting angiogenesis and tumour invasion. While TF shows tumour-enhancing characteristics, its natural inhibitors, TFPI1 and TFPI2, are associated with tumour-suppressing properties [154].

### 3.2. Linc00473

Long intergenic ncRNA 00473 (linc00473) is a lincRNA that has been consistently linked to cancer cell proliferation, survival and metastasis [155,156]. Preeclampsia is a poorly comprehended pathological condition characterized by high blood pressure (hypertension) and proteinuria in the second half of pregnancy [157]. In a study aiming to explore the role of linc00473 in preeclampsia, this lncRNA was found to be downregulated in patients’ placenta, while in vitro, the lncRNA overexpression stimulated trophoblast proliferation. Acting as a molecular guide, Linc00473 was found to bind to lysine-specific demethylase 1 (LSD1), responsible for the demethylation of histone H3 lysine 4 dimethylation (H3K4me2) and histone H3 lysine 9 dimethylation (H3K9me2), thus affecting gene expression and inhibiting the expression of TFPI2 [158]. The role of TFPI2 in preeclampsia is, however, still unclear since both its increased and decreased levels have been reported in preeclamptic patients. According to the current evidence, TFPI2 levels might depend on the expression of glypican-3, a TFPI2-binding protein in placental tissue [159].

### 3.3. AC003092.1

Among glioblastoma patients, the lincRNA AC003092.1 was correlated with increased temozolomide (TMZ) resistance, higher risk of disease relapse and poor prognosis. Even though this study focused on AC003092.1 as a potential therapeutic target for glioblastoma patients, the conducted investigation indicated that this lncRNA regulates TFPI2 expression through the miR-195/TFPI2 axis in glioblastoma. Specifically, by acting as an endogenous CeRNA and, consequently, as a decoy, AC003092.1 prevents miR-195 from targeting TFPI2, thereby increasing TFPI2 expression [160].

### 3.4. AGAP2-AS1

AGAP2 antisense RNA 1 (AGAP2-AS1) is a lincRNA that was first found overexpressed in human NSCLC, being also implicated in other malignant diseases such as colorectal cancer and melanoma [161,162,163]. By interacting with specific RNA-binding proteins, namely enhancers of zeste 2 polycomb repressive complex 2 subunit (EZH2) and LSD1, some lncRNAs can regulate cell phenotypes. The former is a subunit of polycomb repressive complex 2 (PRC2) with catalytic activity, which can suppress gene expression by enhancing histone H3 lysine 27 trimethylation (H3K27me3). As for LSD1, it can repress transcriptional activity through the enzymatic demethylation of histone H3 lysine 4 mono- and dimethylation (H3K4me1/2). In glioblastoma, AGAP2-AS1 acts as a guide and is suggested to inhibit TFPI2 expression through EZH2 and LSD1 binding [164].

### 3.5. MEG8

Long non-coding maternally expressed 8 (MEG8) was shown to be dysregulated in several disorders, such as lung, ovarian and colorectal cancer as well as gestational diabetes mellitus and diabetic nephropathy [165]. A study regarding ischemic heart disease revealed an induction of this gene in patients with this disease. By modulating the expression of TFPI2, which is a known angiogenesis inhibitor, MEG8 is suggested to regulate the angiogenic sprouting [166,167]. In concordance, experiments with HUVECs showed that TFPI2 was five times more expressed after MEG8 silencing. The negative regulation of this lncRNA reduces the inhibitory histone modification H3K27me3 at the TFPI2 promoter, therefore acting as a scaffold. Endothelial function is impaired by MEG8 silencing, suggesting a beneficial role of this lncRNA in preserving cell viability. Knowing that TFPI2 is an angiogenesis inhibitor and the role it plays in the coagulation cascade and extracellular matrix remodelling, the MEG8/TFPI2 axis could be a potential therapeutic target for VTE management [166].

**Table 2:** LncRNAs that regulate TFPI2 expression or activity.

LncRNA	First Author (Country, Year) [Ref]	LncRNA Location ^1^	Disease	Sample/Compartment or Study Model	LncRNA Expression in the Disease	LncRNA Targets	LncRNA Role in TFPI2 Expression
TFPI2AS1	Gao et al. (China, 2017) [153]	7q31-q32	NSCLC	NSCLC tissue and cells	↑	TFPI2	↑
Linc00473	Wu et al. (China, 2018) [158]	6q27	Preeclampsia	Placenta tissues and trophoblast cell lines	↓	LSD1/TFPI2	↓
AC003092.1	Xu et al. (China, 2018) [160]	7q21.3	Glioblastoma	Glioblastoma tissue and cells and mice	↓	MiR-195/TFPI2	↑
AGAP2-AS1	Luo et al. (China, 2019) [164]	12q14.1	Glioblastoma	Glioblastoma tissue and cells and mice	↑	EZH2 and LSD1/TFPI2	↓
MEG8	Kremer et al. (2022, The Netherlands) [166]	14q32.31	Ischemic heart disease	Left ventricular tissues and HUVECs	↓	TFPI2	↑

Abbreviations: HUVECs, human umbilical vein endothelial cells; lncRNA; long non-coding ribonucleic acid; NSCLC, non-small cell lung cancer. ^1^ According to HGNC (HUGO Gene Nomenclature Committee) (https://www.genenames.org/, accessed on 20 May 2023) and Ensembl database (https://www.ensembl.org/index.html, accessed on 20 May 2023). LncRNA or TFPI2 expression: ↓ downregulated and ↑ upregulated.

## 4. Conclusions

Venous thrombogenesis is a common haemostatic disorder associated with substantial morbidity and mortality, particularly in Western countries. Understanding the disease pathophysiology is crucial to improve disease risk stratification, diagnosis and management. Emerging evidence indicates that lncRNAs may have a role in VTE pathogenesis. However, their clinical usefulness as predictive biomarkers for VTE is yet unclear. According to the current review, only a few studies have explored the implications of these ncRNAs in VTE pathways. Moreover, the research repeatability is low, with only MALAT1 and LINC00659 being reported by multiple studies. The lack of external validation in larger cohorts and the limited statistical power of the individual studies are currently the major challenges in this research field. However, as previously mentioned, the first study pinpointing the role of lncRNA in VTE was published in 2019, being that this research field is relatively new. Of note, until now, the studies have only focused on DVT, dismissing a potential role of lncRNAs in PE development. Another limitation of these studies is the fact that the lncRNAs were evaluated in individuals already diagnosed with thrombosis, making it difficult to link these ncRNAs to thrombus formation or thrombus resolution. Meanwhile, multiple lncRNAs have been suggested to modulate the expression of TFPI2, an important anticoagulant. The role of these ncRNAs in VTE pathogenesis should be thus investigated, given the central role of the TF coagulation pathway in thrombosis. Overall, more studies in this field and with real-world VTE patients are required to clarify the clinical use of lncRNAs as VTE biomarkers in an era of minimally invasive methods. As for disease treatment, the use of these ncRNAs is not straightforward. As previously mentioned, the field of lncRNA research is relatively new. A lot of investigation is required to overcome current hurdles before lncRNAs could be translated into effective therapies for VTE patients. The limitations include lncRNAs’ susceptibility to degradation by nucleases present in the bloodstream, the possibility of unintended interactions with off-targets, the possible immune responses that might reduce their effectiveness, and the lack of an established, safe and effective delivery route of lncRNAs to the target site [168,169,170]. Thus, the development of rigorous and more precise investigations using, for instance, animal models should be conducted to better elucidate the roles of these ncRNAs and their therapeutic characteristics and toxicity effects. Using specific lncRNA mimics and inhibitors to change lncRNA expression may be a novel strategy to prevent or treat VTE. In conclusion, this first review on the matter sheds light on the functional significance of lncRNAs in VTE and suggests them as potential biomarkers and therapeutic targets.

## Data Availability

Not applicable.
